# Clinical characteristics and factors related to antibiotic-associated diarrhea in elderly patients with pneumonia: a retrospective cohort study

**DOI:** 10.1186/s12877-021-02267-x

**Published:** 2021-05-17

**Authors:** Yoko Takedani, Tsukasa Nakamura, Noriko Fukiwake, Toshihiro Imada, Junji Mashino, Takeshi Morimoto

**Affiliations:** 1 Department of General Medicine, Shimane Prefectural Central Hospital, Izumo, Japan; 2 Department of Infectious Diseases, Shimane Prefectural Central Hospital, Izumo, Japan; 3 Clinical Education and Research Center, Shimane Prefectural Central Hospital, Izumo, Japan; 4 Department of Clinical Epidemiology, Hyogo College of Medicine, 1-1 Mukogawa, Hyogo 663-8501 Nishinomiya, Japan

**Keywords:** Antibiotic-Associated Diarrhea (AAD), β-Lactamase Inhibitor, Proton Pump Inhibitor (PPI)

## Abstract

**Background:**

Antibiotic-associated diarrhea (AAD) is a common problem among elderly inpatients because many elderly patients are admitted for pneumonia or other conditions that necessitate antibiotic treatment. In the super aging population, more patients are suffering from pneumonia than before, but the incidence or risk factors for AAD among many elderly patients have not been well scrutinized.

**Methods:**

We conducted a retrospective cohort study of elderly patients diagnosed with pneumonia from April 2014 to March 2019 who were admitted to the Department of General Medicine of a Tertiary Care Hospital in Japan. Patients (≥ 65 years of age) who were diagnosed with bacterial pneumonia or aspiration pneumonia and treated with antibiotics were included. We defined AAD by diarrhea with more than three loose or watery stools per day and included patients who had these symptoms for either one day or two or more consecutive days. We also assessed the length of hospital stay and in-hospital mortality. The potential risk factors for AAD included age, sex, body weight, body mass index, smoking, alcohol, activities of daily living (ADL),　comorbidities, vital signs, laboratories, the severity of pneumonia, antibiotic and other medication use.

**Results:**

There were 1,067 patients, the mean age was 83 years, and men accounted for 59 %. β-Lactamase inhibitors were frequently prescribed antibiotics in 703 patients (66 %), and proton pump inhibitors (PPIs) were also commonly administered (48 %). AAD developed in 322 patients (30 %). The multivariate logistic regression model showed that β-lactamase inhibitors (OR 1.43, 95 % CI 1.05–1.95) and PPIs (OR 1.37, 95 % CI 1.03–1.83) were associated with AAD as well as age (OR 1.03 per year, 95 % CI 1.01–1.05).

**Conclusions:**

AAD was common among elderly inpatients with pneumonia, and β-lactamase inhibitors and PPIs were associated with AAD. Strict use of such medication should be considered to decrease the risk of AAD.

## Background

The incidence of pneumonia is high among elderly patients, especially those elder than 75 years [1]. Aspiration pneumonia is especially common among the elderly, and β-lactamase inhibitors are frequently prescribed, which have antibacterial activity against anaerobic bacteria [2]. β-Lactamase inhibitors have been reported to have several adverse effects, and diarrhea is one of the most common adverse effects [3].

Diarrhea occurring after the administration of antibiotics is defined as antibiotic-associated diarrhea (AAD), and *Clostridium difficile* is the most common pathogen of AAD [4]. AAD frequently develops in the elderly population, and antibiotics with a broad spectrum or strong antibacterial activity against anaerobic bacteria were reported as risk factors for AAD [5]. Therefore, elderly patients with pneumonia who are treated with β-lactamase inhibitors have an especially high risk for AAD. Once AAD occurs, it results in not only additional treatment but also an extended hospital stay and extra costs [6–8]. Although several studies have reported factors related to AAD, evidence is scant among the elderly and of the association of β-lactamase inhibitors and AAD. We thus investigated the epidemiology of AAD among elderly patients with pneumonia and explored the clinical characteristics associated with AAD to improve the practice of pneumonia for the elderly considering the risk for AAD.

## Methods

### Study design and patients

We conducted a retrospective cohort study of elderly patients diagnosed with pneumonia from April 2014 to March 2019 who were admitted to the Department of General Medicine of Shimane Prefectural Central Hospital, a tertiary care hospital in Japan. Patients aged 65 years and older who were diagnosed with bacterial pneumonia or aspiration pneumonia and treated with antibiotics were included. The exclusion criteria were patients with (1) viral pneumonia, interstitial pneumonia caused by collagen diseases or other causes, eosinophilic pneumonia, atypical pneumonia, fungal pneumonia and tuberculosis, (2) respiratory infections other than pneumonia such as upper respiratory infections or bronchitis, or (3) diarrhea before admission or transfer from other departments.

We retrieved clinical data, laboratory data and the outcomes of the eligible patients from the Integrated Intelligent Management System (IIMS), which is the unified database that stores the data of electronic medical records, images, ordering system, and other hospital information. The Ethics Review Board of Shimane Prefectural Central Hospital approved this study (approval number: R19-022). Because all data were obtained as a part of routine daily practice, informed consent was waived by the Ethics Review Board of Shimane Prefectural Central Hospital in accordance with the guidelines of the Ministry of Health, Labor and Welfare of Japan.

### Measurements

The retrieved data included age, sex, body weight, body mass index (BMI), history of smoking, alcohol habits, activities of daily living (ADL), comorbidities, vital signs, laboratory date, the severity of pneumonia by CURB 65 score [9], antibiotic use, drug use, microbial test practice. ADL was classified according to ambulatory, bedridden, and whether oral nutrition was provided. Comorbidities contained diabetes mellitus, hypertension, cerebrovascular diseases, ischemic heart disease, heart failure, chronic obstructive pulmonary disease (COPD), chronic kidney disease (CDK), dementia, and cancer. Vital signs contained body temperature, blood pressure, heart rate, and respiratory rate. Laboratory data contained white blood cell, hemoglobin, platelet cell, total protein, albumin, blood urea nitrogen, creatinine, and C-reactive protein on admission. Antibiotics were classified according to whether they contained β-lactamase inhibitors such as sulbactam or tazobactam. If multiple antibiotics were used, the antibiotics that were used first and contributed most to pneumonia treatment were noted. Drug use contained proton pump inhibitor (PPI), immunosuppressant, angiotensin II receptor blocker, angiotensin converting enzyme inhibitor, calcium channel blocker, beta-blocker, psychotropic, and drug for hyperuricemia.

**Fig. 1:**
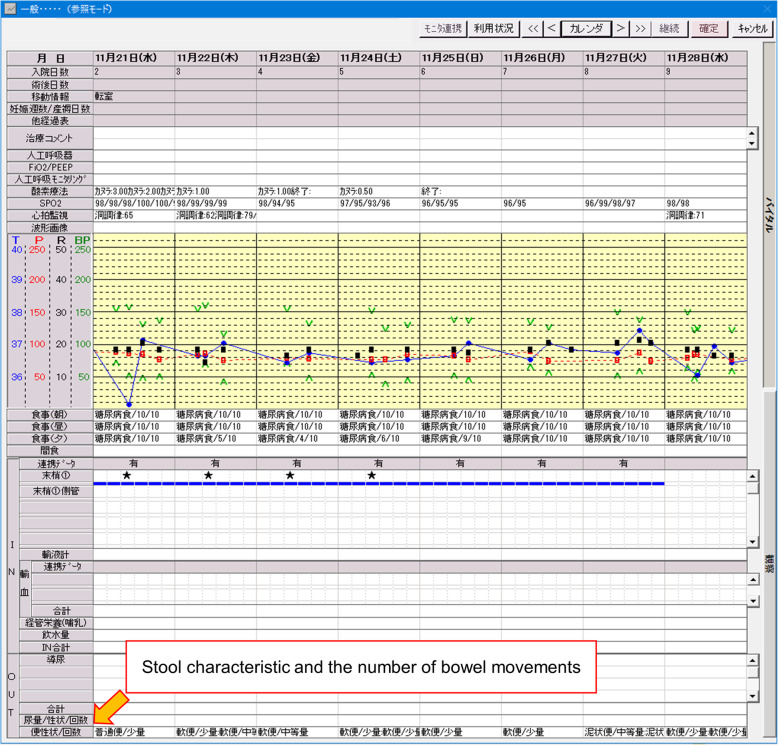
The example of nursing log with diarrhea record. All nurses require to fill out stool characteristicand the number of bowel movements in the Integrated Intelligent ManagementSystem

The primary outcome of this study was AAD. We retrieved stool characteristics and the number of bowel movements from the standard form on the IIMS, which were required to fill out by all nurses in charge (Fig. 1). We defined AAD by diarrhea with more than three loose or watery stools per day and included patients who had these symptoms for either one day [10] or two or more consecutive days [5, 11, 12]. We also assessed the length of hospital stay and in-hospital mortality. If patients were transferred to other departments or hospitals or died during admission, the length of hospital stay was defined as days from admission to discharge from the Department of General Medicine or time of death during admission to the Department of General Medicine. In-hospital mortality was also limited in the Department of General Medicine.

### Statistical analyses

Continuous variables are presented as the mean and standard deviation (SD) or median and interquartile range (IQR), and categorical variables are presented as numbers and percentages. To explore the factors associated with AAD, we compared continuous variables with Student’s *t* test or the Wilcoxon rank-sum test on the basis of the distributions between patients with and without AAD. We compared categorical variables with the χ2 test between patient groups. We then constructed multivariate logistic regression models. We simultaneously included variables with significant univariable associations with AAD in the multivariate models. Associations were expressed with odds ratios (ORs) and 95 % confidence intervals (CIs). All reported *p*-values were two-tailed, and *p*-values < 0.05 were considered statistically significant. All statistical analyses were performed using Stata 14 (StataCorp LLC, Texas) and JMP 12.2 (SAS Institute Inc., Cary, North California).

## Results

### Patient characteristics

There were 1,067 patients included in this study. The mean age was 83 years (SD 8), and the age range was 65–107 years old (Table 1). The number of male patients was 627 (59 %), history of smoking was 468 (44 %), and alcohol habits was 236 (22 %). Mean body weight was 47.3 (SD 11.3), and BMI was 20.0 (SD 6.3). The number of patients who were ambulatory was 682 (64 %), bedridden was 110 (10 %), and on oral nutrition was 1,018 (95 %). The most common comorbidity was hypertension (50 %), followed by dementia (39 %), heart failure (31 %), and cerebrovascular diseases (30 %) (Table 1).

**Table 1:** Patients characteristic and Univariate associations with AAD

Variable	All (*n*=1,067)	AAD (*n*=322)	non-AAD (*n*=745)	*p* values
	no (%) or mean ± SD or median [IQR]			
Age, year	83 ± 8	85 ± 8	83 ± 8	0.0001
Male	627 (59)	180 (56)	447 (60)	0.21
Body weight, kg	47.3 ± 11.3	45.8 ± 10.2	48.0 ± 11.7	0.005
Body mass index, kg/m^2^	20.0 ± 6.3	19.6 ± 3.8	20.0 ± 3.9	0.12
History of smoking	468 (44)	127 (39)	341 (46)	0.056
Alcohol habits	236 (22)	59 (18)	177 (24)	0.045
ADL				
Ambulatory	682 (64)	184 (57)	498 (67)	0.003
Bedridden	110 (10)	35 (11)	75 (10)	0.69
Oral nutrition	1,018 (95)	304 (94)	714 (96)	0.31
Comorbidities				
Diabetes mellitus	297 (28)	85 (26)	212 (29)	0.49
Hypertension	536 (50)	152 (47)	384 (52)	0.19
Cerebrovascular diseases	322 (30)	111 (35)	211 (28)	0.045
Ischemic heart diseases	146 (14)	44 (14)	102 (14)	0.99
Heart failure	325 (31)	114 (35)	211 (28)	0.021
Chronic obstructive pulmonary diseases	254 (24)	64 (20)	190 (26)	0.048
Chronic kidney diseases	64 (6.0)	26 (8.1)	38 (5.1)	0.06
Dementia	419 (39)	142 (44)	277 (37)	0.03
Cancer	253 (24)	79 (25)	174 (23)	0.68
Vital signs				
Body temperature, °C	38.0 ± 0.9	38.0 ± 0.9	38.0 ± 0.9	0.54
Systolic blood pressure, mmHg	150 ± 25	151 ± 25	150 ± 25	0.72
Diastolic blood pressure, mmHg	87 ± 17	87 ± 17	87 ± 18	0.84
Heart rate, /min	103 ± 21	103 ± 22	102 ± 21	0.51
Respiratory rate, /min	29 ± 7	29 ± 7	29 ± 7	0.94
Laboratory data				
White blood cell count, /μL	9,490 [7,260 - 12,850]	9,430 [7,120 - 12,960]	9,540 [7,320 - 12,730]	0.72
Hemoglobin, g/dL	11.8 [10.5 - 13.1]	11.5 [10.4 - 12.8]	11.8 [10.6 - 13.2]	0.001
Platelet cell count, x10^4^/μL	18.9 [14.7 - 24.2]	19.2 [14.9 - 24.4]	18.8 [14.7 - 24.1]	0.63
Total protein, g/dL	6.6 [6.1 - 7.0]	6.5 [6.1 - 7.0]	6.6 [6.1 - 7.0]	0.24
Albumin, g/dL	3.2 [2.8 - 3.6]	3.1 [2.8 - 3.5]	3.3 [2.9 - 3.6]	0.0008
Blood urea nitrogen, mg/dL	18.7 [13.8 - 26.5]	20.0 [14.7 - 27.6]	18.2 [13.5 - 25.7]	0.01
Creatinine, mg/dL	0.76 [0.57 - 1.06]	0.79 [0.57 - 1.10]	0.74 [0.57 - 1.03]	0.26
C-reactive protein, mg/dL	6.91 [2.66 - 12.79]	7.45 [2.69 - 13.25]	6.82 [2.65 - 12.55]	0.74
CURB65				0.06
1	197 (18)	47 (15)	150 (20)	
2	461 (43)	132 (41)	329 (44)	
3	303 (29)	104 (32)	199 (27)	
4	102 (9.6)	38 (12)	64 (8.6)	
5	4 (0.4)	1 (0.3)	3 (0.4)	
Antibiotics				
β-Lactamase inhibitors	703 (66)	233 (72)	470 (63)	0.003
Ampicillin/sulbactam	701 (66)	233 (72)	468 (63)	
Piperacillin/tazobactam	8 (0.8)	4 (1.2)	4 (0.5)	
Non β-Lactamase inhibitors	626 (59)	181 (56)	445 (60)	0.28
Ceftriaxone	390 (37)	107 (33)	283 (38)	
Meropenem	47 (4.4)	25 (7.8)	22 (3.0)	
Macrolides	101 (9.5)	25 (7.8)	76 (10)	
Clindamycin	35 (3.3)	17 (5.3)	18 (2.4)	
Levofloxacin	31 (2.9)	8 (2.4)	23 (3.1)	
Others^a^	189 (18)	67 (21)	122 (21)	
Multiple antibiotics	714 (67)	223 (69)	491 (66)	0.29
Drugs				
Proton pump inhibitors	508 (48)	171 (53)	337 (45)	0.02
Immunosuppressants	363 (34)	102 (32)	261 (35)	0.29
Angiotensin II receptor blockers	117 (11)	44 (14)	73 (9.8)	0.06
Angiotensin converting enzyme inhibitors	54 (5.1)	15 (4.7)	39 (5.2)	0.69
Calcium channel blockers	169 (16)	41 (13)	128 (17)	0.07
Beta-blockers	98 (9.2)	33 (10)	65 (8.7)	0.43
Psychotropics	207 (19)	72 (22)	135 (18)	0.11
Drugs for hyperuricemia	62 (5.8)	22 (6.8)	40 (5.4)	0.35
Sputum culture	954 (89)	289 (90)	665 (89)	0.81
Positive culture	146 (15)	33 (11)	113 (17)	0.03
Hospital course				
AAD	322 (30)			
Duration until AAD, days		6 [3 - 10]		
Duration of AAD, days		4 [2 - 8]		
Length of hospital stay, days	13 [9 - 21]	17 [12 - 29]	11 [8 - 18]	<0.0001
Death	88 (8.2)	22 (6.8)	66 (8.9)	0.27

^a^Ampicillin, Amoxicillin, Cefmetazole, Cefalexin, Cefazolin, Daptomycin, Gentamicin, Micafungin, Metronidazole, Rifampicin, Sulfamethoxazole/Trimethoprim, Vancomycin, AAD: antibiotic-associated diarrhea

The number of patients who used β-lactamase inhibitors was 703 (66 %), and multiple antibiotics was 714 (67 %). The number of patients who took the sputum microbial test was 954 (89 %), of whom 146 (15 %) were detected bacteria (Table 2). Thus, 14 % (146/1067) were considered to have bacterial pneumonia and the rest (86 %) was aspiration or undetermined pneumonia.

**Table 2:** Results of sputum culture

Bacteria (Sputum)	All (*n*=146) N (%)
Gram negative rods	
*Haemophilus influenzae*	33 (23)
*Haemophilus influenzae*	12 (8.2)
*Haemophilus influenzae* (BLNAR)	21 (14)
*Klebsiella pneumoniae*	31 (21)
*Branhamella(Moraxella) catarrhalis*	13 (8.9)
*Escherichia coli*	8 (5.4)
*Escherichia coli*	5 (3.4)
*Escherichia coli* (ESBL)	3 (2)
*Pseudomonas aeruginosa*	7 (4.8)
*Klebsiella oxytoca*	5 (3.4)
Other Gram negative rods	10 (6.9)
*Raoultella planticola*	4 (2.7)
*Acinetobacter baumannii*	1 (0.7)
*Enterobacter aerogenes*	1 (0.7)
*Kluyvera ascorbata*	1 (0.7)
*Pasteurella pneumotropica*	1 (0.7)
*Proteus mirabilis*	1 (0.7)
*Serratia marcescens*	1 (0.7)
Gram negative coccus	
*Neisseria*	1 (0.7)
Gram positive cocci	
*Staphylococcus aureus*	4 (2.7)
Methicillin sensitive *Staphylococcus aureus*	3 (2)
Methicillin resistant *Staphylococcus aureus*	1 (0.7)
*Streptococcus pneumoniae*	30 (21)
*Streptococcus pneumoniae*	29 (20)
Penicillin G insensitive *streptococcus pneumoniae*	1 (0.7)
Other *Streptococcus*	4 (2.8)
*Streptococcus agalactiae* (group B)	1 (0.7)
*Streptococcus anginosus*	1 (0.7)
*Streptococcus* group G	2 (1.4)

*BLNAR* β-lactamase nonproducing Ampicillin resistant, *ESBL* extended spectrum β-lactamase

### Factors associated with AAD

AAD occurred in 322 (30 %) patients. The median duration until AAD was 6 (IQR 3, 10) days, and duration of AAD was 4 (IQR 2, 8) days. Among 322 patients with AAD, *Clostridium difficile* antigen was positive in 14 (4.3 %) patients. The mean age was significantly higher in those who developed AAD (85 vs. 83 years, *p* = 0.0001), and mean body weight and alcohol habits were significantly lower in those who developed AAD (body weight: 45.8 vs. 48.0 kg, *p* = 0.005; alcohol habits: 18 % vs. 24 %, *p* = 0.045). The patients who were ambulatory were less likely to develop AAD (57 % vs. 67 %, *p* = 0.003). There were no significant differences in sex, BMI, history of smoking, bedridden, and oral nutrition between the patients who developed AAD and those who did not. The patients with cerebrovascular diseases, heart failure, and dementia were likely to develop AAD (cerebrovascular diseases: 35 % vs. 28 %, *p* = 0.045; heart failure: 35 % vs. 28 %, *p* = 0.021; dementia: 44 % vs. 37 %, *p* = 0.03), and with COPD were less likely to develop AAD (20 % vs. 26 %, *p* = 0.048). There were no significant associations between other comorbidities and AAD. Median hemoglobin and albumin were significantly lower in those who developed AAD (hemoglobin: 11.5 vs. 11.8 g/dL, *p* = 0.001; albumin: 3.1 vs. 3.3 g/dL, *p* = 0.0008), and median blood urea nitrogen was significantly higher in those who developed AAD (20.0 vs. 18.2 mg/dL, *p* = 0.01). There were no significant differences in vital signs and other laboratory data between the patients who developed AAD and those who did not. There was no significant difference between the CURB 65 score and AAD. The use of β-lactamase inhibitors and PPIs was more frequent among those with AAD than their counterparts (β-lactamase inhibitors: 72 % vs. 63 %, *p* = 0.003; PPIs: 53 % vs. 45 %, *p* = 0.02). There were no significant associations between other drugs and AAD among the elderly patients (Table 1).

The multivariate logistic regression model showed that age (OR 1.03 per year, 95 % CI 1.01–1.05), β-lactamase inhibitors (OR 1.43, 95 % CI 1.05–1.95), and PPIs (OR 1.37, 95 % CI 1.03–1.83) were independently associated with AAD after adjusting for other variables (Table 3).

**Table 3:** Multivariable regression model for AAD

Variable	Odds ratio	95 % confidence interval	*p* values
Age	1.03	1.01–1.05	0.009
Body weight, kg	0.99	0.98–1.01	0.42
Alcohol habits	1.02	0.71–1.48	0.91
Ambulatory	0.84	0.60–1.17	0.30
Cerebrovascular diseases	1.21	0.89–1.64	0.22
Heart failure	1.17	0.85–1.61	0.33
Chronic obstructive pulmonary diseases	0.89	0.62–1.27	0.50
Dementia	1.01	0.74–1.37	0.97
Hemoglobin, g/dL	0.96	0.89–1.05	0.40
Albumin, g/dL	0.81	0.61–1.09	0.17
Blood urea nitrogen, mg/dL	1.01	0.999–1.02	0.07
β-Lactamase inhibitors	1.43	1.05–1.95	0.03
Proton pump inhibitors	1.37	1.03–1.83	0.03

*AAD* antibiotic-associated diarrhea

### Effect of AAD on length of hospital stay and in-hospital mortality

The median length of hospital stay was 13 (IQR 9, 21) days, and in-hospital mortality occurred in 88 (8.2 %) patients. The median length of hospital stay was significantly longer in those who developed AAD (17 vs. 11 days, *p* < 0.0001). However, the in-hospital mortality did not differ between those with and without AAD (6.8 % vs. 8.9 %, *p* = 0.27).

## Discussion

We explored the incidence of AAD and factors associated with AAD in elderly patients with pneumonia. The incidence of AAD was 30 % among elderly patients with a mean age of 83 years. Among such patients, β-lactamase inhibitors were prescribed in 66 % of patients, and β-lactamase inhibitors were significantly associated with AAD. Our study also showed the PPIs were frequently administered among elderly patients, and this class of medication was independently associated with AAD.

The incidences of AAD were lower in previous studies, which were 4.9–9.6 % among hospitalized patients with a mean age of 60–68 years [5, 11]. The 30 % incidence of AAD among inpatients in our study was much higher than those in previous studies, and the differences could partially be due to the large elderly population of our study, with a mean age of 83 years. We showed that the incidence of AAD would be elevated in an older patient population. This finding was also supported by the results of multivariate models of our study, which showed that an increase in age was significantly associated with the risk of AAD. Faced with the superaging population worldwide, the increased incidences of and preventive measures for AAD should be considered. Another explanation of higher incidence of AAD was the differences in the definition of AAD between studies. The definition of AAD was diarrhea with more than three loose or watery stools per day and this definition was as same as previous studies but the duration was at least one day in our study while two days in the previous study [5, 11].

Our study showed several risk and modifiable factors for AAD, namely, β-lactamase inhibitors and PPIs. Some studies found the highest frequencies of AAD in patients treated with broad spectrum penicillins, cephalosporins, and clindamycin [5, 12–14]. Wistrom et al. reported that the highest frequencies of AAD were found in patients treated with tazobactam, which is one of the β-lactamase inhibitors [5], but evidence about the association of β-lactamase inhibitors and AAD was scarce. We therefore classified the antibiotics based on whether they contained β-lactamase inhibitors to clarify the associations between β-lactamase inhibitors and AAD.

Several studies showed that PPIs were associated with AAD or *Clostridium difficile* infection with an OR of 1.98–2.90 [11, 15, 16]. Wong et al. reported that patients who took PPIs had a significantly higher incidence of AAD than those in the non-PPI group [17]. On the other hand, other studies reported no significant association between PPIs and *Clostridium difficile* infection, and some of them targeted elderly patients [10, 18, 19]. Because the association between PPIs and AAD or *Clostridium difficile* infection was reported, associations between PPIs and AAD in our findings should be scrutinized from the perspective of *Clostridium difficile* infection. In addition, collagenous colitis should also be considered in patients with diarrhea on PPIs [20]. Because we defined AAD based on stool characteristics and the number of bowel movements and all enrolled patients had pneumonia, AAD might contain enteritis that was not associated with antibiotics, such as collagenous colitis.

Several potential risk factors for AAD or *Clostridium difficile* infection have been reported, including low ADL [11, 21], tube feeding [4, 6, 14, 22], low serum albumin [15, 23, 24], and renal disease [5, 11, 16, 25]. Tube feeding, low serum albumin and renal disease change the intestinal flora, and furthermore, low serum albumin provokes intestinal edemas [22, 24–27]. These mechanisms promote *Clostridium difficile* infection. Low ADL, tube feeding, and renal disease promote the spread of *Clostridium difficile* spores through care from medical staff and medical procedures [27, 28]. These factors were not independent risk factors for AAD in our study. Although these factors had weak relations with AAD in the univariate models, they were not retained in the multivariate model. Because these factors were related to each other as confounders, these factors were not retained as independent factors. On the other hand, β-lactamase inhibitors and PPIs were still independent adjusting for these factors, and our findings should be considered credible.

β-Lactamase inhibitors were used in 66 % of patients, and PPIs were prescribed in 48 % of patients in our study. We should be aware that many physicians tended to use antibiotics containing β-lactamase inhibitors and prescribe PPIs for elderly patients as a routine practice. We should recognize the risk of AAD due to such medications, especially in combination, and avoid the unnecessary prescription of antibiotics containing β-lactamase inhibitors and PPIs for elderly patients with pneumonia. From the perspective to avoid AAD among elder patients with pneumonia or other infectious diseases, guidelines of antibiotics choice for elder patients should be reconsidered.

Consistent with previous reports [6–8], the median length of hospital stay was significantly longer in those who developed AAD. On the other hand, there was no significant difference between the in-hospital mortality and AAD in our study. Previous studies showed that mortality was significantly higher in those who developed *Clostridium difficile* infection [8, 29, 30]. Because our hospital is an acute care hospital and patients with critical or terminal status tended to transfer to other facilities, our setting was not appropriate to investigate the relationship between AAD and mortality.

There were several limitations. First, our study design was retrospective, and thus we could not obtain information on all potential factors that were not recorded. Therefore, other risk factors might exist, but the information on medication use was well recorded, and the two risk factors in medication use were considered credible. Second, some cases of AAD could have been missed because medical staff could not recognize and record the stool properties and number of bowel movements for all patients. However, the electronic health record was equipped with standard form for diarrhea and the definition of diarrhea was liquid stool greater than 3 times for 24 h in this study. Such missed cases were less likely. In addition, such missing should be happened at random if occurred, the incidence showed the lowest data and the results of multivariable model should be credible. The length of hospital stay or in-hospital mortality could also be underestimated in our study. Third, we could not take into consideration the effects of drugs that induce diarrhea, such as NSAIDs and laxatives or probiotics that prevent or reduce diarrhea. Most of elderly patients often use such drugs or probiotics, but we considered the administration of β-lactamase inhibitors or PPIs was independent to those used such drugs or probiotics. Finally, we did not discriminate the *Clostridium difficile* infection from AAD. Because the detection of *Clostridium difficile* infection at the study hospital was *Clostridium difficile* antigen test which was low sensitivity, the incidence of *Clostridium difficile* infection was underestimated. When we analyze the *Clostridium difficile* precisely, we should culture all stools from AAD for *Clostridium difficile* but this strategy was not realistic in the daily clinical practice. Therefore, we focused on AAD general in this study.

## Conclusions

In elderly patients who were admitted for pneumonia, 30 % developed AAD during the hospital stay, and such patients with AAD had longer hospital stays than those without AAD. The use of β-lactamase inhibitors and PPIs was significantly associated with AAD, and we should pay careful attention to the bowel symptoms in patients who were treated with β-lactamase inhibitors. The use of PPIs should be reevaluated from the perspective of AAD.

## Data Availability

The datasets analyzed during the current study are available from the corresponding author by request.

## Abbreviations

AAD: Antibiotic-associated diarrhea
BMI: Body mass index
ADL: Activities of daily living
COPD: Chronic obstructive pulmonary disease
CDK: Chronic kidney disease
PPI: Proton pump inhibitor
SD: Standard deviation
IQR: Interquartile range
OR: Odds ratio
CI: Confidence intervals
